# Advanced Vibrational Spectroscopy and Bacteriophages Team Up: Dynamic Synergy for Medical and Environmental Applications

**DOI:** 10.3390/ijms25158148

**Published:** 2024-07-26

**Authors:** Magdalena Giergiel, Thulya Chakkumpulakkal Puthan Veettil, Ava Rossetti, Kamila Kochan

**Affiliations:** School of Chemistry, Faculty of Science, Monash University, Clayton, VIC 3800, Australia

**Keywords:** bacteriophages, spectroscopy, infrared, Raman, SERS, AFM-IR, TERS

## Abstract

Bacteriophages are emerging as a promising alternative in combating antibiotic-resistant bacteria amidst the escalating global antimicrobial resistance crisis. Recently, there has been a notable resurgence of interest in phages, prompting extensive research into their therapeutic potential. Beyond conventional microbiology and virology techniques, such as genomics and proteomics, novel phenotypic and chemical characterization methods are being explored. Among these, there is a growing interest in vibrational spectroscopy, especially in advanced modalities such as surface-enhanced Raman spectroscopy (SERS), tip-enhanced Raman spectroscopy (TERS), and atomic force microscopy-infrared spectroscopy (AFM-IR), which offer improved sensitivity and spatial resolution. This review explores the spectrum of uses of vibrational spectroscopy for bacteriophages, including its role in diagnostics, biosensing, phage detection, assistance in phage-based therapy, and advancing basic research.

## 1. Introduction

Bacteriophages, or phages, are viruses that exclusively infect and reproduce within bacteria, often destroying their bacterial hosts [1,2,3]. This unique capability to specifically target and lyse bacteria gained increasing attention, positioning phages as a promising alternative to traditional antibiotic therapies, particularly in the fight against antibiotic-resistant bacteria [1]. While antibiotics have been remarkably effective, the rapid proliferation of resistance among bacterial strains and the lengthy and costly process of developing new antibiotics underscores the urgent need for alternative therapeutic strategies. Leveraging the natural predatory relationship between phages and bacteria, phage therapy presents a viable and innovative solution to this growing global health challenge [1].

Bacteriophages are among the smallest organisms, typically ranging in size from about 20 to 200 nanometers [4,5]. They are composed of a protein capsid that encases their genetic material, which can be either DNA or RNA [1,6]. Each phage particle, known as a virion, is a colloidal entity often exhibiting a dipole moment [2,5]. This feature affects their stability, behavior in solution, and transport properties. The dipole moment can influence aggregation, sedimentation, and interaction with other colloidal particles, which are critical in studying complex fluids, nanomaterials, and targeted drug delivery [5]. The binding mechanism and the resulting bond alterations upon target binding are crucial for biosensing applications; for example, the M13 phage’s binding to sulfur particles in battery technology demonstrates this mechanism [7]. The adaptability of phages was significantly advanced by phage display technology, which connects the phenotype with the genotype, allowing for the selection of target-specific peptides. This achievement was honored by the 2018 Nobel Prize in Chemistry, which was awarded to George P. Smith and Sir Gregory P. Winter [8].

Bacteriophage studies are challenging due to numerous factors, including their genetic diversity, specific host requirements, variable lifecycle strategies, and diverse host–phage interactions [9]. A wide array of sophisticated methods and technologies, such as proteomics, genome sequencing, and high-resolution microscopy (e.g., electron microscopy), are employed in this context [9,10]. However, none of these methods provide a comprehensive phenotypic characterization [9]. Vibrational spectroscopy (VS), particularly with recent advancements in the field, is emerging as a promising new tool for addressing this challenge.

Vibrational spectroscopy, which is rooted in 20th-century scientific exploration, has long been a cornerstone of molecular analysis for various materials [11,12,13,14,15,16]. Traditional techniques, such as Fourier-transform infrared spectroscopy (FTIR) and Raman spectroscopy (RS), provided critical insights into molecular vibrations, enabling the detailed characterization of chemical compositions and structures. FTIR spectroscopy measures the absorption of IR radiation, yielding a spectrum that reflects the molecular fingerprint of the sample [14]. RS, on the other hand, relies on the inelastic scattering of monochromatic light to provide information about vibrational, rotational, and other low-frequency modes in a system [15]. As a non-invasive and label-free method, VS became a powerful tool for examining biological materials, offering several advantages over conventional analytical methods. Numerous reviews in the literature detail its implementations in biological and medical fields [15,16,17]. Advanced modalities like surface-enhanced Raman spectroscopy (SERS), along with combinatory techniques such as atomic force microscopy-infrared spectroscopy (AFM-IR) and tip-enhanced Raman spectroscopy (TERS), broaden the scope of biological applications by improving sensitivity and spatial resolution, compared to conventional vibrational spectroscopy techniques.

The use of VS for molecular characterization and the detection of bacteriophages is opening new avenues in biosensing, nanoscale imaging, mechanistic research, and personalized treatment strategies. Its incorporation in bacteriophage studies is becoming more prevalent and marks a significant evolution in analytical techniques, offering high sensitivity and specificity. This review explores the application of VS to the study of bacteriophages in nanoscience, focusing on its contributions to diagnostics, pathogen detection, phage-based treatment, and basic research.

## 2. Vibrational Spectroscopy Techniques in Bacteriophage Research

### 2.1. Conventional Vibrational Spectroscopy Techniques

**Figure 1:**
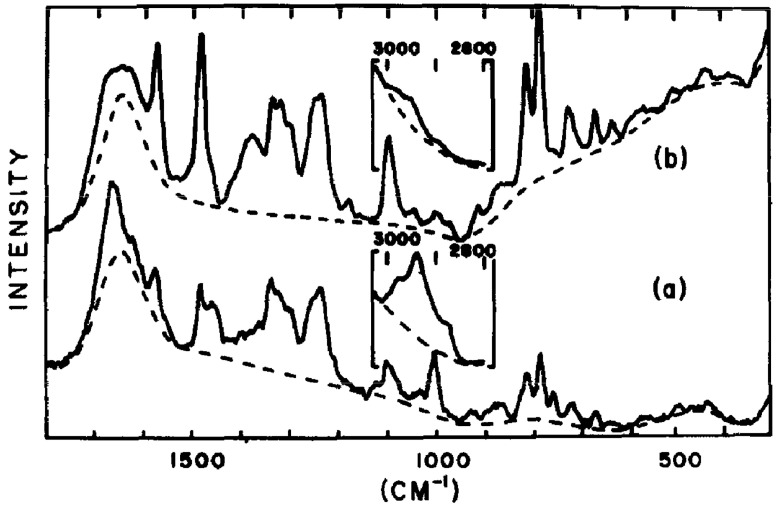
(**a**) First-ever Raman spectrum of R17 bacteriophage in aqueous 0.2M NaCl together with (**b**) Raman spectrum of RNA in aqueous 0.2M NaCl. The solid curves show recorded spectra, while the dashed curves indicate the background scattering from liquid H_2_O. Reproduced with permission from [18].

The first study to utilize vibrational spectroscopy for phages was conducted in 1973 by K.A. Hartman, N. Clayton, and G.J. Thomas, Jr. on the R17 virus and its RNA [18]. The authors demonstrated the first-ever Raman spectrum of the R17 phages, which revealed characteristic vibrations of nucleotide residues and protein capsomers (Figure 1). Following this, G.J. Thomas Jr. et al. used Raman spectroscopy to examine other bacteriophages. They provided detailed insights into their structure and stability by demonstrating the conformations of coat-protein and RNA components [19,20,21,22]. These studies showed that the RNA within the virion displays a highly ordered secondary structure, stabilized by the capsid, which prevents significant structural changes at high temperatures, particularly in high ionic strength environments. These pioneering efforts introduced Raman spectroscopy as a viable method for examining bacteriophage structures in aqueous environments. This indicates its broader application in bacteriophage research and for other nucleoproteins, such as ribosomes and chromosomes. Nowadays, Raman spectroscopy is recognized as a promising novel approach for the detection of bacteriophages [23].

Apart from its historical significance in structural analysis, RS offers powerful capabilities for the detection and discrimination of different phages based on their vibrational spectra. Researchers can distinguish between different types of bacteriophages by analyzing the unique Raman fingerprints of each phage, which are characterized by specific vibrational modes of their protein and nucleic acid components. This discriminatory capability is particularly valuable in fields such as microbiology and virology, in which the precise identification of phage species is crucial for understanding their roles in bacterial infections and developing targeted therapeutic and diagnostic strategies. The ability of RS to provide a rapid, label-free, and non-destructive analysis makes it an ideal tool for these applications, offering insights into phage diversity and behavior that are essential for advancing research in microbial ecology and biotechnology. A concise description of Raman spectroscopy application in studying viruses and microorganisms, including bacteriophages, is presented in [24], focusing on studies of the chemical composition and interactions critical for pharmaceutical and medical applications.

Raman spectroscopy offers several advantages over conventional IR, making it better suited for bacteriophage studies, including minimal interference from liquid water, versatility in sample states, and rapid spectroscopic acquisition times [25]. Although there are few examples of the use of FTIR in the literature, its applicability is more limited, mainly due to lower spatial resolution and strong water interference. Furthermore, developments in Raman instrumentation and data analysis expanded its application to large biomolecular assemblies. The sensitivity and selectivity of RS make it invaluable for examining viral assembly pathways, structural changes under varying conditions, and interactions at the molecular level. Notably, it is important to highlight that accurate Raman vibrational band assignments are essential for interpreting structural details and understanding viral protein recognition and assembly processes.

### 2.2. SERS

Implementing advanced techniques, such as SERS, can significantly enhance RS sensitivity and selectivity. SERS is a robust analytical technique widely explored in biosensing due to its high sensitivity, real-time response, and capacity for molecular fingerprinting [26,27,28]. In detail, SERS exploits two primary mechanisms to enhance Raman scattering signals. Firstly, when incident light matches the LSPR frequency of nanoparticles, it generates intense electromagnetic fields at their surfaces. This enhancement of the electromagnetic field significantly boosts the Raman scattering cross-section of adjacent molecules, resulting in much stronger signals than traditional Raman spectroscopy. Secondly, chemical enhancement occurs through interactions between adsorbed molecules and the metal surface, involving charge transfer processes that modify the Raman scattering behavior. Out of the vibrational spectroscopy techniques, SERS is the most commonly used in bacteriophage research and is often applied to study the molecular composition and interactions of bacteriophages, particularly their unique structural and functional properties.

### 2.3. AFM-IR and TERS

Recent advancements in the field of vibrational spectroscopy, in particular, the introduction of combinatory techniques such as AFM-IR and TERS, expanded research capabilities, enabling the study of smaller organisms and structures. AFM-IR is a rapidly emerging technique that utilizes the tip of an AFM probe to detect and measure the thermal expansions of samples’ surfaces triggered by the absorption of short pulses of infrared light [29]. By integrating AFM and IR techniques, the inherent limitations of each are overcome, enabling the acquisition of chemical composition information (inaccessible via AFM alone) and achieving a spatial resolution as fine as 20 nm, thus surpassing the optical diffraction limitation of conventional IR [30]. To date, AFM-IR has been utilized for the subcellular imaging and spectroscopic characterization of bacterial and mammalian cells [30,31,32,33] and for studying intracellular molecular compounds [29] and viruses, including bacteriophages [34,35]. Similarly, TERS provides nanospectroscopy benefits by combining RS and scanning probe microscopy, enabling greater sensitivity and spatial resolution than conventional RS. Although AFM-IR and TERS share scanning probe microscopy chassis, they differ in their physical principles; AFM-IR measures light absorbed by the sample, while TERS measures scattered light, yielding two distinct yet complementary spectra.

## 3. Spectroscopy for Diagnosis, Pathogen Detection, and Biosensing

### 3.1. Direct Phage Detection

VS has been applied for the direct detection of phages in both environmental [36,37] and clinical samples [37,38]. The work by L. J. Goeller and M. R. Riley from 2007 focused on utilizing RS and SERS for the direct detection of and differentiation between bacteriophages MS2 and PRD1 and *E. coli* bacteria in environmental samples [36]. This approach demonstrated distinct spectral fingerprints for each microorganism; MS2 exhibited strong spectral features at 1667, 1320 cm^−^^1^, and 1088 cm^−^^1^, while PRD1 had considerably more intense bands at 1607 and 1249 cm^−^^1^ due to the presence of protein moieties, compared to the *E. coli*. Both MS2 and PRD1 showed detection limits of approximately 10^9^ PFU/mL, while the detection limit for *E. coli* was reported as 10^6^ cells/mL. Notably, SERS enhanced the signal intensity by 10^3^-fold, although it showed typically higher variability in band features.

In a parallel effort [38], researchers aimed to advance virus detection and identification using RS integrated with machine learning techniques. They demonstrated the method’s viability by analyzing saliva spiked with P1 phage, M13 phage, and Lambda phage. By training a supervised convolutional neural network, they successfully detected and classified different phage types amidst significant spectral noise. This innovative approach highlights its capability for rapid, accurate, and cost-effective virus detection, overcoming inherent challenges such as low signal intensity and noise interference.

FTIR spectroscopy, combined with chemometrics, was also proven effective for phage discrimination [37]. This technique was successfully applied to differentiate five closely related *Salmonella enterica* serotype *Enteritidis* phage types (PT1, PT1b, PT4b, PT6, and PT6a). The study analyzed intact cells and outer membrane protein (OMP) extracts, finding that OMP extracts provided better separation between phage types. The OMP-based model achieved 100% accuracy in differentiating PT1-PT1b, PT4b, and PT6-PT6a groups. Local models further improved discrimination, with 98.3% accuracy for PT1–PT1b and 86% for PT6–PT6a. The robustness of the method was confirmed using isolates from different phage types. FTIR spectroscopy, particularly when using OMP extracts, demonstrated significant potential for the rapid and accurate discrimination of *Salmonella* phage types, indicating its usefulness in diagnostic labs and food safety assessments.

### 3.2. Phage-Based Detection of Bacteria

#### 3.2.1. Clinical Aspect: Toward the Diagnosis of Bacterial Infections

The ability to rapidly and accurately detect bacterial pathogens is a longstanding challenge and critical necessity in healthcare, food safety, water quality, and beyond. The presence of bacterial pathogens is associated with conditions like sepsis, which is a life-threatening syndrome characterized by the body’s severe response to infection that is recognized as one of the leading causes of death globally [39,40]. In 2020, there were 48.9 million cases of sepsis and 11 million sepsis-related deaths globally, comprising 20% of all global deaths, with nearly half occurring in children under 5 years old [40]. In addition, the rapidly increasing prevalence of antimicrobial resistance (AMR) is further exacerbating the problem and underscoring the urgency of effective pathogen detection [1,41]. One of the explored innovative alternatives for bacterial detection is based on the use of bacteriophages, taking advantage of their specificity and ability to selectively target bacterial pathogens and often incorporating RS or SERS due to their inherent capability to detect single microbes [42,43,44,45].

In 2016, a study presented a novel method for detecting *Pseudomonas aeruginosa* using a phage-capture system coupled with micro-RS [42]. By functionalizing commercial latex beads with engineered phage clones, this method concentrated bacteria from clinical samples, significantly amplifying the Raman signal without the need for selective media or extensive biochemical tests and achieving rapid detection within an hour. The development of a phage-based assay for detecting bacterial pathogens in blood using Raman spectroscopy, with a focus on improving sepsis diagnosis, was later reported by [43]. The study employed M13 phage clones specific to *Staphylococcus aureus*, *Pseudomonas aeruginosa*, and *Escherichia coli* attached to magnetic beads for pathogen capture and concentration, achieving a limit of detection of 10 CFU/7 mL of blood. This approach highlighted the integration of magnetic separation with RS as an efficient method for early bacterial identification, offering expedited diagnostic outcomes with reduced cost compared to traditional methods.

**Figure 2:**
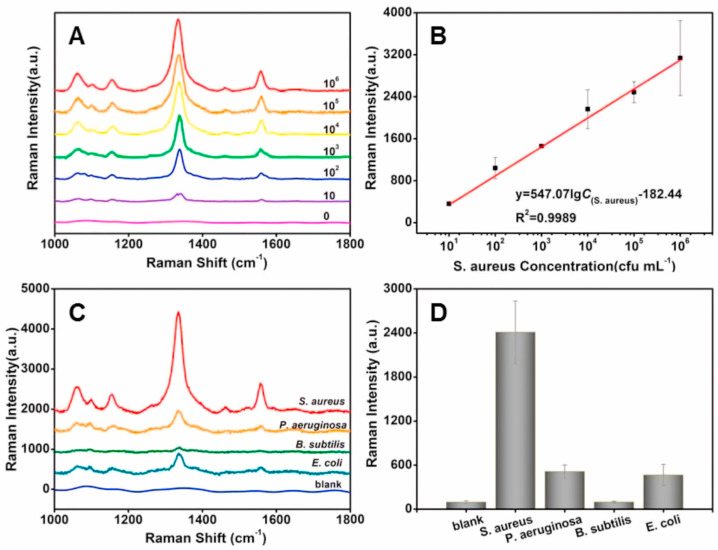
(**A**) The recorded SERS spectra of M13-SERS probe in the presence of various concentrations of *S. aureus*: 10^6^, 10^5^, 10^4^, 10^3^, 10^2^, 10, and 0 CFU/mL; (**B**) the calibration curve between Raman intensity and the concentration of *S. aureus*; (**C**) SERS spectra of M13-SERS probe in the presence of various bacteria at the concentration of 10^6^ CFU/mL; (**D**) Raman intensity of the M13-SERS probe at 1331 cm^−1^ in the presence of *S. aureus*, *B. subtilis*, *E. coli*, and *P. aeruginosa*. Each spectrum is the average of three independently collected spectra. Reproduced with permission from [44].

Similarly, Wang et al. presented a novel strategy for the selective detection and inactivation of *S. aureus* using a SERS nanoprobe based on the M13 phage [44]. The M13-SERS probe was created by growing gold nanoparticles (AuNPs) in situ on the M13 phage surface, followed by subsequent modification with 5,5′-dithiobis-(2-nitrobenzoic acid) as the SERS-active molecule. When introduced, the SERS probe allowed the M13 phage to selectively bind to *S. aureus*, facilitating the attachment of AuNPs to the bacterial surface. As shown in Figure 2, a clear decrease in the SERS signal is observed with the decreasing concentration of *S. aureus*. Importantly, the signal remains distinguishable from the control, even at a concentration as low as 10 CFU/mL for *S. aureus*.

The analytical performance of bacteriophages has been compared to that of antibodies, which are commonly used as analyte-specific agents in lateral flow immunoassay test strips [46,47]. In fact, bacteriophages could serve as an alternative to antibodies in lateral flow assays for bacteria, as they provide comparable sensitivity, specificity, and accuracy. Ihah et al. developed an F5-4 phage-based lateral flow assay, which specifically targets *Salmonella enteritidis*, and demonstrated significantly higher SERS responses for *S. enteritidis* compared to other bacteria types, including *Bacillus subtilis*, *Micrococcus luteus*, *Escherichia coli*, and *Salmonella typhimurium* [46]. In another example, Stambach et al. developed a novel SERS–lateral flow immunochromatographic platform utilizing anti-A511-conjugated SERS nanoparticles combined with A511 bacteriophage amplification for the rapid and specific detection of *L. monocytogenes*. The approach is capable of detecting progeny A511 at concentrations as low as 6 × 10^6^ PFU/mL [48].

In a diagnostic context, bacteriophages are also used as biotemplates for constructing high-performance SERS-active nanostructures with significant SERS enhancement effects and signal reliability. Franco et al. examined the use of engineered molecular networks between bacteriophages and gold nanoparticles as SERS probes for the rapid detection of bacterial cells, using *P. aeruginosa* as a model system [49]. The study by Jeon et al. investigated the ability of bacteriophage MS2 to act as a biotemplate or molecular scaffold for a raspberry-shaped assembly of gold nanoparticles [50]. The close assembly of nanoparticles by bacteriophage MS2 and a rigid molecular linker, captopril, enabled a significant enhancement of the plasmon coupling effect, thereby generating a high near-field enhancement between nanometer-sized interparticle gaps. Sokullu et al. used the M13 bacteriophage as a biotemplate to establish precise spacing between small AuNPs (3–13 nm) and utilized SERS to investigate the strength of plasmon coupling within the gaps of the AuNPs assembled on the M13 template [51]. These promising strategies could be deployed to construct high-performance SERS-active nanostructures for various molecular sensing and analysis applications.

#### 3.2.2. Environmental Pathogens and Food Safety

Vibrational spectroscopy is emerging as a pivotal tool in food safety. It demonstrates potential in both bacterial and viral detection and is driving recent explorations across various sectors to address critical challenges in the food supply chain [52]. In this context, bacteriophages are gaining attention as targeted agents for pathogen detection [53,54,55,56], similar to previously discussed clinical adaptation.

Rippa et al. demonstrated the application of Tbilisi bacteriophages in conjunction with high-performance quasicrystal-patterned nanocavities for the detection of *Brucella abortus* in milk samples [57]. *Brucella abortus* is a Gram-negative bacterium known to infect cattle and lead to brucellosis. The phages covalently immobilized on surfaces and acted as effective bio-receptors, while the nanocavity structures facilitated significant Raman enhancement for SERS. In a related study, the group employed a different SERS-based approach for *Brucella* detection, utilizing novel multilayer octupolar metastructures combined with self-assembled monolayers and immobilized Tbilisi bacteriophages [58]. This method enabled sensitive measurements at the single-cell level, allowing for a rapid and reliable identification of pathogenic bacteria. The development of multilayer octupolar metastructures is particularly noteworthy for its dual functionality in detecting local refractive index changes and enhancing Raman signals with high sensitivity. These findings set the foundation for future investigations aiming to immobilize various bacteriophages for the selective identification of diverse bacterial pathogens at lower concentrations, promising advancements in biosensing technologies for food safety and beyond.

Recently, Jeon and collaborators reported the first label-free SERS detection of *Erwinia amylovora* using the bacteriophage phiEaSP1 specific to *E. amylovora* [59]. *E. amylovora* is one of the most extensively studied plant pathogens, which is notorious for causing fire blight—a severe bacterial infection primarily affecting apple and pear trees [56]. The approach that was used highlighted a novel application in which specific bacteriophages facilitate SERS analysis without the need for traditional peak assignment in spectral interpretation. Instead, the method relies on monitoring temporal changes in SERS signals, demonstrating its potential for real-time bacterial detection and environmental monitoring applications.

Bacteriophages were also found to be useful in monitoring the presence of various pathogens in water systems [60] using FTIR spectroscopy combined with an electrodeposition process. This method utilizes an electrically charged optical element to capture viruses and other charged materials onto an attenuated total reflectance (ATR) crystal. The FTIR measurement then provides quantitative and qualitative information about the deposited material. Initial studies demonstrated the ability to differentiate among three proteins and two viruses, including bacteriophages, based on their spectral signatures. This approach offers significant advantages over traditional molecular recognition techniques, as it allows for the capture and analysis of a wide variety of materials directly from water samples [60]. The IR measurement, using the evanescent wave, penetrates only 1 µm from the ATR crystal surface, ensuring high specificity and minimizing interference from suspended particles. The method showed successful virus and bacteriophage deposition within 5 min, making it feasible for near-real-time monitoring. It also allowed for the partial reversibility of the deposition process by reversing the voltage polarity and enabling the reuse of the crystal surface. The approach has the potential for continuous, automated monitoring of municipal drinking water systems, providing the rapid and accurate detection and discrimination of pathogens, including bacteriophages.

Collectively, these studies underscore vibrational spectroscopy’s versatility and potential in advancing rapid and specific microbial detection, offering promising implications for environmental monitoring [59], food safety applications [57,58,59,61], and even wastewater management [62].

### 3.3. Phage-Based Detection of Cancer Cells and Viruses

The introduction and advancements in phage display technology paved the way for the development of various phage-based biosensors [63,64,65,66], including identifying specific types of cancer cells. Lentini et al. report utilizing molecular networks composed of entire bacteriophage structures, which display specific peptides directly assembled with silver nanoparticles [67]. This configuration serves as a new SERS probe for the identification of U937 cells using a 9-merpVIII M13 phage display library, which is screened against U937 to identify a clone that selectively recognizes these cells [67].

Bacteriophages were also used for the identification of SARS-CoV-2 infections [68,69], in which they were utilized as SERS probes. Single-chain Fv (scFv) recombinant antibody fragments were conjugated to magnetic nanoparticles and SERS nanotags to create a phage-SERS platform. This assay was able to detect 4.1 × 10^4^ genomes/mL, which is (10−100)-fold lower than the viral loads typically found in infectious individuals. Additionally, the assay offered highly sensitive virus detection and a rapid sample-to-result time of 30 min [69]. Phage display technology was also used recently to create a nonpathogenic model for SARS-CoV-2 [70]. By fusing the nucleocapsid protein of SARS-CoV-2 with the M13 bacteriophage’s capsid protein, the researchers developed a model that successfully expressed the target protein, verified through molecular techniques. The phage-based model was evaluated for qPCR quantification, showing strong interactions with anti-N-protein antibodies, making it a viable substitute for the pathogenic virus in research. Notably, the study employed ATR- FTIR spectroscopy to classify and identify the phage model, demonstrating effective sample differentiation using principal component analysis–linear discriminant analysis (PCA–LDA). This highlights the potential of FTIR in developing virus monitoring systems and safely studying highly pathogenic viruses like SARS-CoV-2.

## 4. Spectroscopy as an “Assistance Tool” in Phage-Based Treatment Strategies

**Figure 3:**
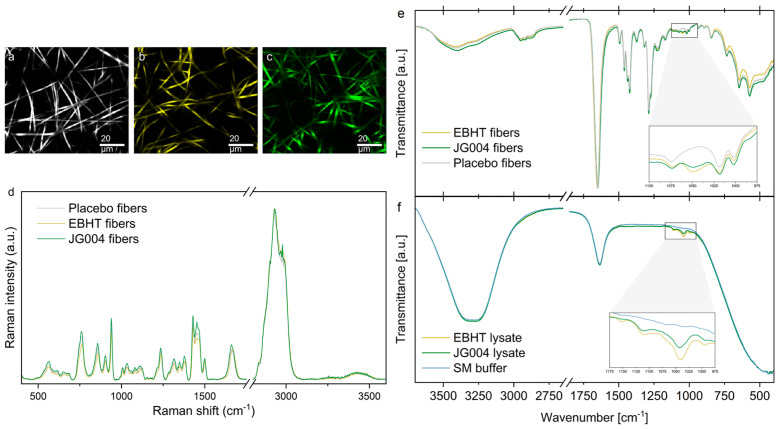
(**a**) Raman false-color images of placebo fibers; (**b**) fibers encapsulating the *Staphylococcus* phage EBHT; (**c**) fibers the encapsulating *Pseudomonas* phage JG004 and (**d**) the corresponding Raman fiber spectra. Raman spectra of bacteriophage-loaded fibers did not show a substantial difference from placebo fibers, indicating intact polymer shell structures shielding the biological cargo in the core. (**e**) Infrared spectra of fiber mats and (**f**) the corresponding pure components. A distinct peak between 1040 and 1050 cm^−1^ was found to be exclusively present in samples loaded with bacteriophages, confirming the successful bacteriophage encapsulation. Reproduced with permission from [73].

The growing popularity of bacteriophages stems from their high efficiency and specificity in targeting bacterial cells, positioning them as promising alternatives for therapy [71,72]. In this context, spectroscopy emerges as a valuable assisting tool, offering the potential to expedite the selection of suitable bacteriophage candidates and evaluate diverse phage-based strategies. An interesting example was provided by Kielholz et al. [73], who explored the development of bacteriophage-loaded electrospun fiber mats tailored for treating antibiotic-resistant bacterial infections caused by *S. aureus* and *P. aeruginosa*. These fibers were engineered with a protective polymer sheath encapsulating active bacteriophages in the core using a coaxial electrospinning technique. The fibers exhibited consistent morphology, robust mechanical properties suitable for wound care, and compatibility with human skin cells. Researchers used both Raman and FTIR spectroscopies to characterize the bacteriophage mats (Figure 3). This platform technology highlights the sustained antimicrobial efficacy of encapsulated bacteriophages over extended periods and positions itself as a promising approach for phage therapy in clinical settings.

Ning et al. investigated the potential of 21 *Salmonella* phages as an alternative approach to combat biofilms [74]. Their study demonstrated that phage cocktail treatment, particularly featuring phage CW1, significantly reduced *Salmonella* cells within biofilms by disrupting extracellular polymeric substances, as confirmed through Raman analysis. This method holds promise in effectively sterilizing biofilms, emphasizing the role of Raman spectroscopy as a valuable tool for optimizing phage-based treatments, which play a role in clinical settings and as a preventive measure for food safety.

## 5. Spectroscopy for Mechanistic Insights: Phage–Bacteria and Phage–Virus Interactions

Spectroscopy, with its inherent ability to provide chemical composition in a label-free and non-invasive manner, has the potential to significantly contribute to understanding bacteriophage mechanisms and interactions. As such, it is increasingly implemented in various contexts of bacteriophage research. We have included a summary of the types of phages and bacteria investigated using vibrational spectroscopy in Supplementary Table S1.

Monsees et al. [75] employed Raman microspectroscopy to study the biochemical alterations within bacterial and archaeal cells post-phage infection. By analyzing single-cell spectra before and after virus addition, significant differences were identified, particularly in *Pseudomonas syringae* infected with dsRNA phage phi6 and *Bacillus subtilis* infected with dsDNA phage phi29. This method highlights a spectral ratio as indicative of virocells, demonstrating its potential for studying host responses at a molecular level.

**Figure 4:**
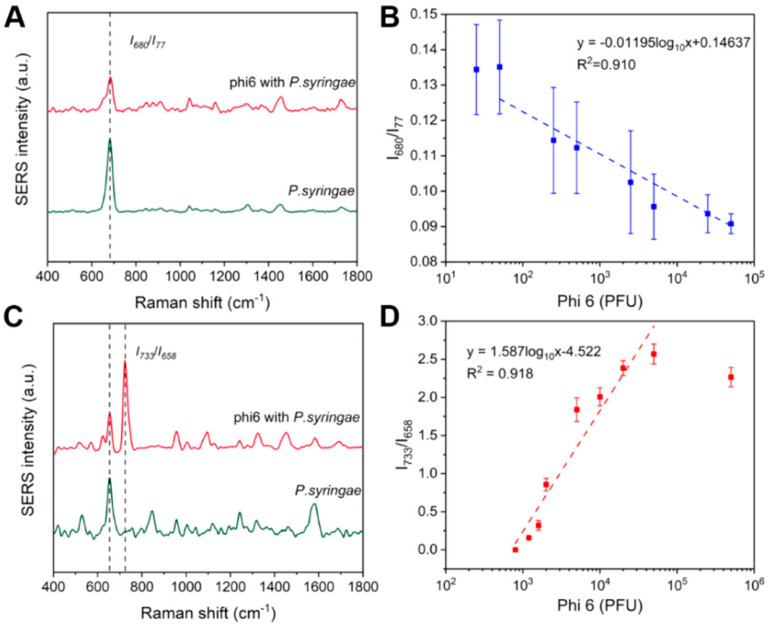
(**A**) SERS spectra of volatile metabolites of uninfected *P. syringae* and Phi6 (50,000 PFU)-infected *P. syringae*; (**B**) linear relationship between I_680_/I_77_ and the logarithm of Phi6 concentration (500−50,000 PFU); (**C**) SERS spectra of nonvolatile substances of *P. syringae* and Phi6 (10,000 PFU)-infected *P. syringae*; and (**D**) linear relationship between I_733_/I_658_ and logarithm of Phi6 concentration (1200−50,000 PFU). Reproduced with permission from [76].

Wang et al. explored the utility of SERS for detecting and quantifying bacterial metabolites, as well as monitoring the growth of lytic bacteriophage Phi6 [76]. Phi6, used as a model for enveloped viruses such as SARS-CoV-2 and influenza, was studied in the context of infecting *P. syringae* as a host system. The findings revealed that bacterial metabolic activity is significantly suppressed in the presence of lytic bacteriophage Phi6. In particular, the LB medium exhibited strong bands at 733 cm^−1^ (adenine or phosphatidylserine) and 658 cm^−1^ (guanine), with varying intensities correlating with initial Phi6 concentrations, allowing for quantification based on the peak ratio (I_733_/I_658_). Figure 4 shows the SERS spectra of volatile metabolites of uninfected *P. syringae* and various concentrations of Phi6 (50,000 PFU and 10,000 PFU)-infected *P. syringae*. Using support vector machine (SVM) classification on Phi6-infected and uninfected samples, the authors achieved an impressive overall accuracy of 93%. Furthermore, decreased intensities of bands at 526 cm^−1^ (S−S disulfide stretch in proteins), 842 cm^−1^ (glucose), 1092 cm^−1^ (C−C skeletal and C−O−C stretching from glycosidic linkages), and 1582 cm^−1^ (phenylalanine) indicated lower concentrations of the extracellular metabolites in phage-infected bacterial samples compared to controls.

Mehmood et al. characterized the phage-sensitive *S. aureus* bacteria exposed to bacteriophages of the *Siphoviridae* family using silver nanoparticles as SERS substrates [77]. The bands at 575 cm^−1^ (C–C skeletal mode), 620 cm^−1^ (phenylalanine), 649 cm^−1^ (tyrosine, guanine, ring breathing), 657 cm^−1^ (guanine, COO deformation), 728–735 cm^−1^ (adenine, glycosidic ring mode), and 796 cm^−1^ (tyrosine, C–N stretching) were identified as indicative of the bacterial degradation accompanying the ongoing replication of bacteriophages. The multivariate classification approach, partial least squares–discriminant analysis (PLS–DA), demonstrated a sensitivity and specificity of 94.47% and 98.61%, respectively. Similarly, in another study, phage-susceptible and resistant strains of methicillin-resistant *S. aureus* (MRSA) exhibited characteristic SERS spectral features. These features are relevant to the biochemical changes that occur as a result of developing bacteriophage resistance, as well as the unique characteristics related to each individual strain. The PLS–DA model achieved 100% specificity, 100% accuracy, and 99.8% sensitivity for SERS spectral data sets of bacterial cell pellets [78].

Phage efficiency was also investigated by Olszak et al., who explored the relationship between the efficacy of *Pseudomonas* phages and the phenotypic properties of *P. aeruginosa* strains [79]. The study involved 28 lytic phages tested against 121 *P. aeruginosa* isolates, including mucoid strains from cystic fibrosis patients. An FTIR analysis focused on the carbohydrate spectral window, revealing significant correlations between phage susceptibility and the chemical composition of the bacterial isolates. While most cystic fibrosis-derived strains were susceptible to multiple phages, some exhibited complete resistance. Notably, phages PA5oct and KT28 demonstrated strong lytic activity against a substantial portion of the strains. An in vitro analysis underscored the influence of bacterial properties, such as biofilm formation, mucosity, twitching motility, and biochemical profiles, on phage susceptibility [79]. In vivo tests using the *Galleria mellonella* model corroborated the in vitro findings, showing that certain cystic fibrosis isolates resistant to phages in vitro were more susceptible to phage cocktails in vivo. The study demonstrated that FTIR spectroscopy is a valuable tool for understanding the biochemical diversity of *P. aeruginosa* strains and its impact on phage efficacy, directly linking biochemical composition with phage effectiveness. These insights underscore spectroscopy’s potential as a powerful tool for assessing phage therapy potential, particularly in the treatment of drug-resistant pathogens and chronic infections.

Garg and team developed an in situ spatiotemporal SERS approach to monitor dynamic, heterogeneous interactions between viruses and bacterial networks using bacteriophage virus Phi6-infected *Pseudomonas syringae* as a model system [80]. They employed novel Au−SiO_2_−Au nanolaminated plasmonic crystals comprising dense and uniformly distributed SERS hotspots to facilitate the in situ spatiotemporal SERS measurements of live *P. syringae* biofilms and compared spatiotemporal biochemical changes in the biological matrix due to the phage−bacteria interaction. An analysis of the phage dosage-dependent response using PCA–LDA found that the temporal dynamics of different biomolecules, including amino acids, nucleic acids, and lipids observed in the SERS spectra, reflect the virus-specific alteration of the metabolism of host bacteria. This approach could be employed in various applications, such as phage-based anti-biofilm therapy development and continuous pathogenic virus detection.

Furthermore, laser tweezers Raman spectroscopy (LTRS) has emerged as a powerful tool for studying dynamic cellular processes [81,82]. Study [81] employed LTRS to observe and quantify real-time cellular responses of *E. coli* cells undergoing lysis induced by external agents like egg white lysozyme and temperature changes with bacteriophage λcI857. The technique revealed distinct Raman spectral changes associated with each lysis method, elucidating molecular dynamics, such as ribosome unfolding and molecular release. Moreover, Raman tweezers were utilized in a recent study to investigate bacteriophage–host interactions within *S. aureus* strains infected with virulent phage JK2 and temperate phage 80α [82]. This approach enabled the real-time monitoring of molecular changes in infected cells, detecting replicating phages shortly after infection onset. Key Raman spectral features attributed to nucleotides, phosphodiester bonds, and heme were crucial in distinguishing between infected and uninfected cells within minutes. This highlights the potential of Raman tweezers for advancing personalized phage therapy and biotechnological applications, offering insights into phage–host dynamics.

Additionally, alternative approaches to Raman spectroscopy broadened its applications beyond traditional methods, encompassing diverse bacterial and viral research studies. For example, one team [83], reported the study of low-frequency vibrational modes (≤20 cm^−1^) of bacteriophage M13 in aqueous environments. By identifying specific Raman modes, like the axial torsion mode at 8.5 cm^−1^ within the phage protein coat, the study highlighted Raman spectroscopy’s utility in nanotechnology, especially for monitoring virus functionalization and assembly processes with nanomaterials.

## 6. Nanospectroscopy for Bacteriophage Imaging

In all cell imaging, a high lateral resolution is needed to identify and characterize the sample [34]. However, the lateral resolution of conventional microspectroscopy techniques ranges in the microns, which is insufficient for accurately and sensitively identifying bacteriophages’ nanochemical and structural features [29]. Optical nanospectroscopy techniques, such as AFM-IR, offer a solution for a more rapid method of the label-free identification and structural characterization of viruses, including bacteriophages, without losing sensitivity and spatial resolution.

Dazzi et al. demonstrated the successful application of AFM-IR to create chemical maps that facilitate the localization of phages within bacteria [34]. These maps are based on the DNA absorption band at 1080 cm^−1^, reflecting the high concentration of DNA/RNA in viruses, which constitutes approximately 70% of a bacteriophage’s mass. The authors also illustrated AFM-IR’s capability to document various stages of phage infection, ranging from uninfected bacteria (empty capsid) to partially invaded cells and, ultimately, to cells largely invaded by mature phages. Similarly, Khanal et al. used AFM-IR to detect and analyze bacteriophages in spray-dried powder [35]. The study showed that AFM-IR provided unparalleled resolution for spectroscopic imaging, allowing for the detection of bacteriophages and the precise assessment of their protein composition within the powder.

**Figure 5:**
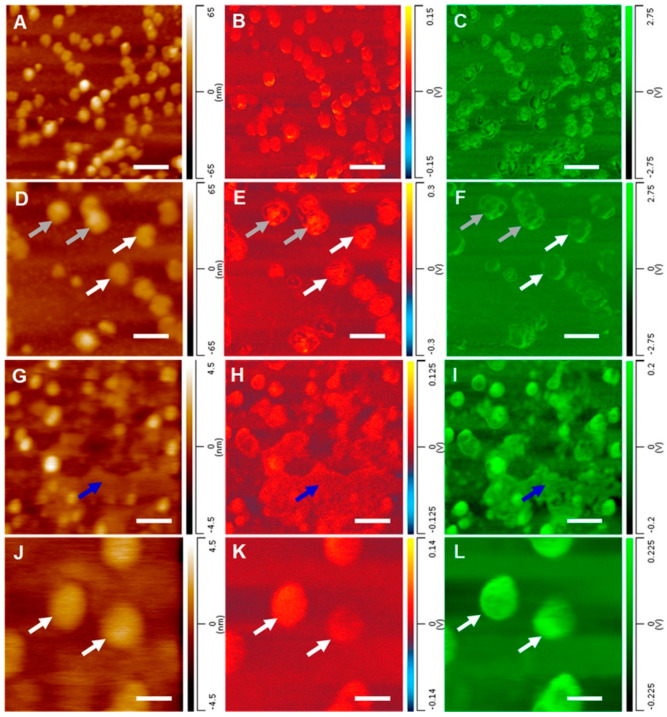
AFM-IR imaging of individual, (**A**–**F**) HSV-1, and (**G**–**L**) MS2 virions. Chemical maps of protein (panels (**B**,**E**,**H**,**K**)) and nucleic acid (panels (**C**,**F**,**I**,**L**)) components of viral particles reveal their exterior and interior components. Scale bar = 600 nm (panels (**A**–**C**)), 200 nm (panels (**G**–**I**)), 300 nm (panels (**D**–**F**)), and 68 nm (panels (**J**–**L**)). Viral particles are shown by white and gray arrows; residual cell debris is shown by a blue arrow. Reproduced with permission from [29].

TERS is another advanced spectroscopic modality, offering improved spatial resolution compared to its conventional counterpart (RS). It can detect and characterize viruses and phages and resolve both double-stranded and single-stranded DNA, achieving single-base resolution for the latter [84,85]. TERS measurements additionally provide information regarding the surface morphology of a virus and crucial surface-located proteins that facilitate bacterial infection [86]. However, since TERS primarily probes surface characteristics, it is typically employed in conjunction with AFM-IR as a dual nanospectroscopy method, offering insights into both surface protein composition and the composition of the viral interior [29]. Dou et al. used this combination of techniques to examine the structural character of individual virions of the herpes simplex type 1 virus and MS2 bacteriophage [29]. AFM-IR probed the exterior (protein capsid) and interior structure (nucleic acid) of the individual viruses (Figure 5), while TERS enabled the determination of the secondary structure of the surface-bound proteins and their amino acid composition. Therefore, although TERS is less commonly applied in viral studies than AFM-IR, both techniques are effective methods for detecting, imaging, and measuring viruses such as bacteriophages, whether alone or in combination.

## 7. Future Outlook and Conclusions

Bacteriophages present a promising avenue, particularly as an alternative therapy in the battle against antibiotic-resistant bacteria [1,3]. Despite being discovered before antibiotics, phages were overshadowed by penicillin due to their perceived complexities and specificity [1]. The escalating AMR crisis drives the recent resurgence of interest in phages, necessitating the search for new effective treatment strategies, improved diagnostics, and better monitoring [3]. The increasing number of publications in the field of bacteriophage research over the past decade highlights this renewed focus. In this context, vibrational spectroscopy is emerging as a powerful platform for molecular characterization.

Phages exhibit a natural selectivity toward bacteria while maintaining a non-hostile relationship with mammalian cells, making them excellent candidates for bacterial targeting. The ability of VS to characterize molecular compositions and, especially, to discriminate between DNA and RNA signatures makes it highly compatible with the use of bacteriophages as labels for pathogens, providing a promising new avenue in pathogen detection. This synergy is especially favorable for developing new diagnostic tools, in which the rapid acquisition of spectra using techniques like ATR-FTIR offers high-speed diagnostics. The advancement of chemometrics-assisted complex data analysis further enhances the sensitivity of detecting minor alterations, making the combination of phage-based targeting and spectroscopic techniques a powerful strategy for pathogen detection.

The implications of this approach extend beyond healthcare and clinical diagnostics. The One Health concept recognizes the interdependence of human, animal, plant, and ecosystem health, emphasizing the need for a holistic pathogen detection and treatment strategy. Phage–VS methodologies can be applied to all these samples, thus playing a crucial role in environmental and food safety.

Furthermore, bacteriophages offer extensive potential as biosensors that greatly exceed their innate ability to target bacteria due to the phage display technology [5,66]. Initially introduced for presenting polypeptides on lysogenic filamentous bacteriophages, phage display is now efficiently used to display a great variety of peptides, proteins, and antibodies [5]. Phages are easily manipulated genetically to incorporate foreign DNA, are stable under diverse conditions, and can remain active in various environments [66]. These properties, combined with the diversity and scalability of phage technology, make them ideal carriers for displaying foreign genes, thereby expanding their utility to detect and combat various diseases, such as cancer or COVID-19.

A particularly promising application of phage display technology lies in phage-based vaccines, presenting a compelling alternative to traditional vaccination methods [64,87,88,89]. Unlike conventional vaccines, phages are non-infectious to eukaryotes, chemically stable, cost-effective for large-scale production, and easily transportable [89]. They hold significant potential as vaccines against multidrug-resistant pathogens and in addressing the challenges of cancer prevention, for which, to date, there is still no effective vaccine [90]. Efforts are currently underway to explore the possibility of a phage-based COVID-19 vaccine [91]. Further research is needed to fully assess their impact on mammalian cells and elucidate the mechanisms underlying their immune response elicitation [92]. Vibrational spectroscopy, with its capacity for studying immune signaling at the cellular level [93,94], could significantly contribute to advancing this research.

Phage therapy remains the most compelling application of bacteriophages, especially against resistant strains. As naturally selective and highly efficient agents, phages offer an encouraging therapeutic alternative to antibiotics. There are several examples of successful phage-based therapy in the literature [72,95,96,97,98]. However, challenges such as proper phage selection and understanding their detailed mechanisms of action and impact on mammalian cells need to be addressed. VS could significantly contribute to this area by speeding up the selection of phage cocktails, monitoring their effects, and providing comprehensive chemical profiling in a label-free, spatially localized manner. This capability extends to studies of living cells under physiological conditions (RS-based techniques).

The ability to profile chemical interactions and characterize cellular responses through spectroscopic methods provides critical insights into phage mechanisms and their therapeutic potential, as evidenced by the growing volume of research utilizing these techniques. These studies range from characterizing different phages and their interactions with bacterial hosts to linking phage efficiency with the chemical profile of bacterial strains. Such knowledge can improve the understanding of phage efficiency against resistant strains and their effects on biofilms and mammalian cells. Techniques like SERS, TERS, and AFM-IR offer significant signal enhancement and single-phage spatial resolution, presenting attractive opportunities for basic research.

The future of bacteriophage research and its integration with vibrational spectroscopy holds immense promise. Continued advancements in spectroscopic techniques, coupled with AI-driven data analytics, can refine our ability to characterize phage interactions at a molecular level. Future research should prioritize enhancing our understanding of phage mechanisms, optimizing phage selection for therapy against resistant pathogens, and elucidating their broader ecological impacts. Furthermore, exploring novel applications in fields such as agriculture, environmental monitoring, and personalized medicine can expand the utility of phages beyond infectious disease management. Ultimately, harnessing the synergy between phage biology and spectroscopic technology represents a frontier in biotechnological innovation with far-reaching implications for healthcare, agriculture, and environmental sustainability.

## Supplementary Materials

The following supporting information can be downloaded at: https://www.mdpi.com/article/10.3390/ijms25158148/s1.
