# Supplementary material for: Advanced Vibrational Spectroscopy and Bacteriophages Team Up: Dynamic Synergy for Medical and Environmental Applications
Source: Int J Mol Sci. 2024 Jul 26;25(15):8148. doi: 10.3390/ijms25158148 (PMC11311505; doi:10.3390/ijms25158148)
Supplement: Supplementary file 1 [file ijms-25-08148-s001.zip › ijms-3104105-supplementary.pdf]

Supplementary Materials

Advanced Vibrational Spectroscopy and Bacteriophages Team Up: Dynamic Synergy for Medical and Environmental Applications

Magdalena Giergiel[1], Thulya Chakkumpulakkal Puthan Veettil[1], Ava Rossetti[1] and Kamila Kochan[1,*]

[1] School of Chemistry, Faculty of Science, Monash University, Victoria, 3800, Australia
* Correspondence: kamila.kochan@monash.edu

Article	Type of phage	Type of bacteria
Chemical mapping of the distribution of viruses into infected bacteria with a photothermal method [1]	T5	*Escherichia coli*
High Resolution Nanoscale Probing of Bacteriophages in an Inhalable Dry Powder Formulation for Pulmonary Infections [2]	Staphylococcal phage S83	NA
Tip-Enhanced Raman Spectroscopy of Combed Double-Stranded DNA Bundles [3]	λ-phage virus	NA
Tip-Enhanced Raman Imaging of Single-Stranded DNA with Single Base Resolution [4]	phage ssDNA (M13mp18)	NA
Machine learning approach for label-free rapid detection and identification of virus using Raman spectra [5]	P1, M13, Lambda	NA
A simple and fast method for discrimination of phage and antibiotic contaminants in raw milk by using Raman spectroscopy [6]	Φ231-X9, Φ231-X21, Φ231-X23, ΦB3-X1, ΦB3-X11, 1ΦB3-X13, Φ709-X4, Φ1B3	*Streptococcus thermophilus*
Bacteriophage-loaded functional nanofibers for treatment of P. aeruginosa and S. aureus wound infections [7]	EBHT phages, JG004 phages	*Staphylococcus aureus* and *Pseudomonas aeruginosa*
Secondary structure and interactions of the packaged dsDNA genome of bacteriophage P22 investigated by Raman difference spectroscopy [8]	P22	NA
Structural Studies of P22 Phage, Precursor Particles, and Proteins by Laser Raman spectroscopy [9]	P22	NA
Biofilm removal mediated by Salmonella phages from chicken-related sources [10]	CW1, CW11, M4, M10	*Salmonella*
Phage-based assay for rapid detection of bacterial pathogens in blood by Raman spectroscopy [11]	M13	*Staphyloccocus aureus*, *Pseudomonas aeruginosa* and *Escherichia coli*
Analysis of Bacteriophage–Host Interaction by Raman Tweezers [12]	JK2 Myoviridae family and a temperate phage 80α of the Siphoviridae family	*Staphylococcus aureus*
Laser Tweezers Raman Spectroscopy Potential for Studies of Complex Dynamic	λcI857	*Escherichia coli*

Cellular Processes: Single Cell Bacterial Lysis [13]		
Applications of Raman Spectroscopy to Virology and Microbial Analysis [14]	P22, PRD1, Pf1&fd, MS2	various
Label-Free Raman Microspectroscopy for Identifying Prokaryotic Virocells [15]	phi29, phi6	*Pseudomonas syringae*, *Bacillus subtilis*, *Methanosarcina mazei* (archaea)
Observation of the low frequency vibrational modes of bacteriophage M13 in water by Raman spectroscopy [16]	M13	NA
Rapid detection of Pseudomonas aeruginosa by phage-capture system coupled with micro-Raman spectroscopy [17]	P9b phage from M13	*Pseudomonas aeruginosa*
Surface-Enhanced Raman Spectroscopy of Bacterial Metabolites for Bacterial Growth Monitoring and Diagnosis of Viral Infection [18]	Phi6	*Pseudomonas syringae*
Precisely Controlled Three-Dimensional Gold Nanoparticle Assembly Based on Spherical Bacteriophage Scaffold for Molecular Sensing via Surface-Enhanced Raman Scattering [19]	MS2	NA
Bio-hybrid gold nanoparticles as SERS probe for rapid bacteria cell identification [20]	P9b phage clone	*Pseudomonas aeruginosa*
Surface-enhanced Raman spectroscopy for the characterization of bacterial pellets of Staphylococcus aureus infected by bacteriophage [21]	Siphoviridae family	*Staphylococcus aureus*
Label-Free Surface-Enhanced Raman Scattering Detection of Fire Blight Pathogen Using a Pathogen-Specific Bacteriophage [22]	phiEaSP1	fire blight bacteria *Erwinia amylovora*
Discrimination of Bacteria and Bacteriophages by Raman Spectroscopy and Surface-Enhanced Raman Spectroscopy [23]	MS2 and PRD1	*Escherichia coli*
Rapid Detection of Listeria by Bacteriophage Amplification and SERS-Lateral Flow Immunochromatography [24]	A511	*Listeria monocytogenes*
M13 phage-based nanoprobe for SERS detection and inactivation of Staphylococcus aureus [25]	M13	*Staphylococcus aureus*
Plasmonic Enhancement of Two-Photon Excitation Fluorescence by Colloidal Assemblies of Very Small AuNPs Templated on M13 Phage [26]	M13	NA
Replacement of antibodies with bacteriophages in lateral flow assay of Salmonella Enteritidis [27]	F5-4	*Salmonella enteritidis*
In Situ Spatiotemporal SERS Measurements and Multivariate Analysis of Virally Infected Bacterial Biofilms Using Nanolaminated Plasmonic Crystals [28]	Phi6	*Pseudomonas syringae*
SERS Biosensor Based on Engineered 2D-Aperiodic Nanostructure for In-Situ Detection of Viable Brucella Bacterium in Complex Matrix [29]	Tb phages	*Brucella abortus*
Octupolar Metastructures for a Highly Sensitive, Rapid, and Reproducible Phage-	Tb phages	*Brucella abortus*

Based Detection of Bacterial Pathogens by Surface-Enhanced Raman Scattering [30]		
Bacillus thuringiensis Cells Selectively Captured by Phages and Identified by Surface Enhanced Raman Spectroscopy Technique [31]	BtCS33 phages	*Bacillus thuringiensis*/*Bacillus anthracis*
Phage–AgNPs complex as SERS probe for U937 cell identification [32]	9-mer pVIII M13 phage	U937 cancer cells
Colorimetric and surface-enhanced Raman scattering dual-mode lateral flow immunosensor using phage-displayed shark nanobody for the detection of crustacean allergen tropomyosin [33]	phagedisplayed shark nanobody	NA
Construction, Characterization, and Application of a Nonpathogenic Virus-like Model for SARS-CoV-2 Nucleocapsid Protein by Phage Display [34]	M13	NA
Integrated Capture and Spectroscopic Detection of Viruses [35]	MS2	NA
Application of Fourier Transform Infrared Spectroscopy and Chemometrics for Differentiation of Salmonella enterica Serovar Enteritidis Phage Types [36]	Salmonella Enteritidis phage types, phage type 1 (PT1), PT1b, PT4b, PT6, and PT6a	*Salmonella enterica*
In vitro and in vivo antibacterial activity of environmental bacteriophages against Pseudomonas aeruginosa strains from cystic fibrosis patients [37]	environmental Pseudomonas phages (28 different)	*Pseudomonas aeruginosa*

Supplementary Table S1. An overview of various research articles focused on the application of VS in studying bacteriophages. The table highlights the diversity of bacteriophage species and bacterial strains investigated using VS, showcasing its utility in microbiological and virological research applications.

[1] Dazzi, A., Prazeres, R., Glotin, F., Ortega, J.M., Al-Sawaftah, M., de Frutos, M. Chemical mapping of the distribution of viruses into infected bacteria with a photothermal method. Ultramicroscopy. 2008, 108, 635-41.

[2] Khanal, D., Chang, R.Y.K., Morales, S., Chan, H.-K., Chrzanowski, W. High Resolution Nanoscale Probing of Bacteriophages in an Inhalable Dry Powder Formulation for Pulmonary Infections. Analytical Chemistry. 2019, 91, 12760-7.

[3] Najjar, S., Talaga, D., Schué, L., Coffinier, Y., Szunerits, S., Boukherroub, R., et al. Tip-Enhanced Raman Spectroscopy of Combed Double-Stranded DNA Bundles. The Journal of Physical Chemistry C. 2014, 118, 1174-81.

[4] He, Z., Han, Z., Kizer, M., Linhardt, R.J., Wang, X., Sinyukov, A.M., et al. Tip-Enhanced Raman Imaging of Single-Stranded DNA with Single Base Resolution. Journal of the American Chemical Society. 2019, 141, 753-7.

[5] Alexander, R., Uppal, S., Dey, A., Kaushal, A., Prakash, J., Dasgupta, K. Machine learning approach for label-free rapid detection and identification of virus using Raman spectra. Intelligent Medicine. 2023, 3, 22-35.

[6] Acar-Soykut, E., Tayyarcan, E.K., Boyaci, I.H. A simple and fast method for discrimination of phage and antibiotic contaminants in raw milk by using Raman spectroscopy. J Food Sci Technol. 2018, 55, 82-9.

[7] Kielholz, T., Rohde, F., Jung, N., Windbergs, M. Bacteriophage-loaded functional nanofibers for treatment of P. aeruginosa and S. aureus wound infections. Scientific Reports. 2023, 13, 8330.

[8] Aubrey, K.L., Casjens, S.R., Thomas, G.J., Jr. Secondary structure and interactions of the packaged dsDNA genome of bacteriophage P22 investigated by Raman difference spectroscopy. Biochemistry. 1992, 31, 11835-42.
[9] Thomas, G.J., Jr., Li, Y., Fuller, M.T., King, J. Structural studies of P22 phage, precursor particles, and proteins by laser Raman spectroscopy. Biochemistry. 1982, 21, 3866-78.
[10] Ning, Z., Zhang, L., Cai, L., Xu, X., Chen, Y., Wang, H. Biofilm removal mediated by Salmonella phages from chicken-related sources. Food Science and Human Wellness. 2023, 12, 1799-808.
[11] De Plano, L.M., Fazio, E., Rizzo, M.G., Franco, D., Carnazza, S., Trusso, S., et al. Phage-based assay for rapid detection of bacterial pathogens in blood by Raman spectroscopy. J Immunol Methods. 2019, 465, 45-52.
[12] Pilát, Z., Jonáš, A., Pilátová, J., Klementová, T., Bernatová, S., Šiler, M., et al. Analysis of Bacteriophage–Host Interaction by Raman Tweezers. Analytical Chemistry. 2020, 92, 12304-11.
[13] Chen, D., Shelenkova, L., Li, Y., Kempf, C.R., Sabelnikov, A. Laser Tweezers Raman Spectroscopy Potential for Studies of Complex Dynamic Cellular Processes: Single Cell Bacterial Lysis. Analytical Chemistry. 2009, 81, 3227-38.
[14] Harz, M., Stöckel, S., Ciobotă, V., Cialla, D., Rösch, P., Popp, J. Applications of Raman Spectroscopy to Virology and Microbial Analysis. In: Emerging Raman Applications and Techniques in Biomedical and Pharmaceutical Fields, Matousek, P., Morris, M.D. Eds., Springer Berlin Heidelberg, Berlin, Heidelberg, 2010, pp. 439-63.
[15] Monsees, I., Turzynski, V., Esser Sarah, P., Soares, A., Timmermann Lara, I., Weidenbach, K., et al. Label-Free Raman Microspectroscopy for Identifying Prokaryotic Virocells. mSystems. 2022, 7, e01505-21.
[16] Tsen, K.T., Dykeman, E.C., Sankey, O.F., Lin, N.T., Tsen, S.W., Kiang, J.G. Observation of the low frequency vibrational modes of bacteriophage M13 in water by Raman spectroscopy. Virol J. 2006, 3, 79.
[17] Lentini, G., Franco, D., Fazio, E., De Plano, L.M., Trusso, S., Carnazza, S., et al. Rapid detection of Pseudomonas aeruginosa by phage-capture system coupled with micro-Raman spectroscopy. Vibrational Spectroscopy. 2016, 86, 1-7.
[18] Wang, W., Kang, S., Vikesland, P.J. Surface-enhanced Raman spectroscopy of bacterial metabolites for bacterial growth monitoring and diagnosis of viral infection. Environmental science & technology. 2021, 55, 9119-28.
[19] Jeon, M.J., Ma, X., Lee, J.U., Roh, H., Bagot, C.C., Park, W., et al. Precisely controlled three-dimensional gold nanoparticle assembly based on spherical bacteriophage scaffold for molecular sensing via surface-enhanced Raman scattering. The Journal of Physical Chemistry C. 2021, 125, 2502-10.
[20] Franco, D., De Plano, L., Rizzo, M., Scibilia, S., Lentini, G., Fazio, E., et al. Bio-hybrid gold nanoparticles as SERS probe for rapid bacteria cell identification. Spectrochimica Acta Part A: Molecular and Biomolecular Spectroscopy. 2020, 224, 117394.
[21] Mehmood, N., Akram, M.W., Majeed, M.I., Nawaz, H., Aslam, M.A., Naman, A., et al. Surface-enhanced Raman spectroscopy for the characterization of bacterial pellets of Staphylococcus aureus infected by bacteriophage. RSC advances. 2024, 14, 5425-34.
[22] Jeon, Y., Lee, S., Vu, N.T., Kim, H., Hwang, I.S., Oh, C.-S., et al. Label-Free Surface-Enhanced Raman Scattering Detection of Fire Blight Pathogen Using a Pathogen-Specific Bacteriophage. Journal of Agricultural and Food Chemistry. 2024.
[23] Goeller, L.J., Riley, M.R. Discrimination of Bacteria and Bacteriophages by Raman Spectroscopy and Surface-Enhanced Raman Spectroscopy. Applied Spectroscopy. 2007, 61, 679-85.
[24] Stambach, N.R., Carr, S.A., Cox, C.R., Voorhees, K.J. Rapid detection of Listeria by bacteriophage amplification and SERS-lateral flow immunochromatography. Viruses. 2015, 7, 6631-41.
[25] Wang, X.-Y., Yang, J.-Y., Wang, Y.-T., Zhang, H.-C., Chen, M.-L., Yang, T., et al. M13 phage-based nanoprobe for SERS detection and inactivation of Staphylococcus aureus. Talanta. 2021, 221, 121668.

[26] Sokullu, E., Pinsard, M., Zhang, J., Plathier, J., Kolhatkar, G., Blum, A.S., et al. Plasmonic enhancement of two-photon excitation fluorescence by colloidal assemblies of very small AuNPs templated on M13 phage. Biomacromolecules. 2020, 21, 2705-13.
[27] Ilhan, H., Tayyarcan, E.K., Caglayan, M.G., Boyaci, İ.H., Saglam, N., Tamer, U. Replacement of antibodies with bacteriophages in lateral flow assay of Salmonella Enteritidis. Biosensors and Bioelectronics. 2021, 189, 113383.
[28] Garg, A., Nam, W., Wang, W., Vikesland, P., Zhou, W. In situ spatiotemporal SERS measurements and multivariate analysis of virally infected bacterial biofilms using nanolaminated plasmonic crystals. ACS sensors. 2023, 8, 1132-42.
[29] Rippa, M., Castagna, R., Sagnelli, D., Vestri, A., Borriello, G., Fusco, G., et al. SERS biosensor based on engineered 2D-aperiodic nanostructure for in-situ detection of viable Brucella bacterium in complex matrix. Nanomaterials. 2021, 11, 886.
[30] Rippa, M., Castagna, R., Pannico, M., Musto, P., Borriello, G., Paradiso, R., et al. Octupolar metastructures for a highly sensitive, rapid, and reproducible phage-based detection of bacterial pathogens by surface-enhanced Raman scattering. Acs Sensors. 2017, 2, 947-54.
[31] Almaviva, S., Palucci, A., Aruffo, E., Rufoloni, A., Lai, A. Bacillus thuringiensis Cells Selectively Captured by Phages and Identified by Surface Enhanced Raman Spectroscopy Technique. Micromachines (Basel). 2021, 12.
[32] Lentini, G., Fazio, E., Calabrese, F., De Plano, L.M., Puliafico, M., Franco, D., et al. Phage–AgNPs complex as SERS probe for U937 cell identification. Biosensors and Bioelectronics. 2015, 74, 398-405.
[33] Jiao, S., Chen, X., He, Z., Wu, L., Xie, X., Sun, Z., et al. Colorimetric and surface-enhanced Raman scattering dual-mode lateral flow immunosensor using phage-displayed shark nanobody for the detection of crustacean allergen tropomyosin. Journal of Hazardous Materials. 2024, 133821.
[34] Wu, Y., Liu, B., Liu, Z., Zhang, P., Mu, X., Tong, Z. Construction, Characterization, and Application of a Nonpathogenic Virus-like Model for SARS-CoV-2 Nucleocapsid Protein by Phage Display. Toxins. 2022, 14, 683.
[35] Vargas Crystal, A., Wilhelm Allison, A., Williams, J., Lucas, P., Reynolds Kelly, A., Riley Mark, R. Integrated Capture and Spectroscopic Detection of Viruses. Applied and Environmental Microbiology. 2009, 75, 6431-40.
[36] Preisner, O., Guiomar, R., Machado, J., Menezes, J.C., Lopes, J.A. Application of Fourier Transform Infrared Spectroscopy and Chemometrics for Differentiation of <i>Salmonella enterica</i> Serovar Enteritidis Phage Types. Applied and Environmental Microbiology. 2010, 76, 3538-44.
[37] Olszak, T., Zarnowiec, P., Kaca, W., Danis-Wlodarczyk, K., Augustyniak, D., Drevinek, P., et al. In vitro and in vivo antibacterial activity of environmental bacteriophages against Pseudomonas aeruginosa strains from cystic fibrosis patients. Applied Microbiology and Biotechnology. 2015, 99, 6021-33.
